# *In vitro* Activation Prior to Transplantation of Human Ovarian Tissue: Is It Truly Effective?

**DOI:** 10.3389/fendo.2019.00520

**Published:** 2019-08-02

**Authors:** Marie-Madeleine Dolmans, Florence Cordier, Christiani A. Amorim, Jacques Donnez, Catherine Vander Linden

**Affiliations:** ^1^Pôle de Recherche en Gynécologie, Institut de Recherche Expérimentale et Clinique (IREC), Université Catholique de Louvain, Brussels, Belgium; ^2^Department of Gynecology, Cliniques Universitaires Saint Luc, Université Catholique de Louvain, Brussels, Belgium; ^3^Société de Recherche pour l'Infertilité, Brussels, Belgium

**Keywords:** slow-freezing, vitrification, ovarian tissue transplantation, hippo pathway, Akt pathway, PTEN inhibitors, SCID mice, *in vitro* activation

## Abstract

**Research Question:** What are the true benefits, if any, of disrupting the Hippo signaling pathway and stimulating the Akt pathway in xenotransplanted human ovarian tissue using an *in vitro* activation (IVA) approach?

**Design:** Human ovarian tissue was retrieved from 18 young patients by laparoscopy and grafted to 54 severe combined immunodeficient mice. The experiment was conducted using fresh ovarian tissue (group I; *n* = 6 women), slow-frozen-thawed ovarian tissue (group II; *n* = 6 women), and vitrified-warmed ovarian tissue (group III; *n* = 6 women). Slow-freezing and vitrification procedures were performed according to Gosden's and Kawamura's protocols, respectively. The tissue (fresh, slow-frozen, and vitrified) was fragmented into small cubes (1 × 1 × 1 mm) to disrupt the Hippo signaling pathway and cultured or not in IVA medium for 48 h with Akt stimulators (PI3K stimulator and PTEN inhibitor), before being transplanted to the mice. All the grafts were maintained for 28 days.

**Results:** (1) *Follicular density:* Follicular density decreased in all groups after transplantation, most significantly in the vitrification group. Culture with IVA had no impact. (2) *Follicle activation*: Addition of PI3K stimulator and PTEN inhibitor for 48 h prior to grafting did not significantly change the proportion of primordial follicles in any of the groups (fresh, slow-frozen, or vitrified tissue) compared to 48 h of control culture without these molecules. Particularly, vitrification and culture in IVA medium yielded no benefits in terms of growing follicle percentages or follicle proliferation rates. The large proportion of growing follicles in the vitrified tissue group after grafting may have been responsible for the higher rate of atresia.

**Conclusion:** We were unable to demonstrate any significant benefits of cutting ovarian tissue into small cubes and applying IVA with Akt stimulators. The association of vitrification and transplantation was actually found to be the most deleterious combination with respect to the follicle reserve, and even worse when culture with Akt stimulators was performed.

## Introduction

Human folliculogenesis is a complex process regulated by endocrine hormones and paracrine and autocrine factors of oocyte and granulosa cell (GC) origins (1). Primordial follicles reside as a large and quiescent population in the ovary. While most primordial follicles remain in a state of arrest due to dormancy factors, selected primordial follicles initiate growth, developing to the primary stage under the control of serine/threonine kinases through two signaling pathways: protein kinase B (Akt) and mammalian target of rapamycin (mTOR) (1). The Akt pathway, or phosphoinositide-3-kinase (PI3K)-Akt, is a signal transduction pathway that promotes survival, growth, and proliferation in response to oocyte- and GC-derived factors (1, 2). Once activated, primordial follicles start growing and advance to primary and secondary stages under the influence of paracrine factors and, later, follicle-stimulating hormone (FSH).

By contrast, development of preantral follicles is curbed by the inhibitory Hippo signaling pathway (1, 3), so disruption of Hippo signaling promotes secretion of downstream growth factors capable of stimulating follicle growth (1, 2). Disruption of this pathway (which occurs with ovarian tissue fragmentation), combined with stimulation of Akt signaling by treatment with phosphatase and tensin homolog (PTEN) inhibitors and/or PI3K stimulators, was indeed shown to promote growth of pre-antral follicles (2, 4–6). This combined method, known as *in vitro* activation (IVA), was proposed by Kawamura and Suzuki to promote development of antral follicles in women with a diminished ovarian reserve (2, 6). Two live births have so far been reported after ovarian tissue vitrification, IVA, and reimplantation in Japan (2, 5–7). In 2016, Zhai et al. reported a live birth in a series of 14 patients with pre-mature ovarian insufficiency (POI) after removal of one ovary, 2 days of IVA, and reimplantation beneath the serosa of one of the fallopian tubes (8).

The general objective of this study was therefore to evaluate the true impact of disrupting the Hippo signaling pathway, associated with activation of the Akt pathway, on human ovarian tissue in a xenotransplantation model. Fresh, slow-frozen, and vitrified human ovarian tissues were all subjected to analysis.

## Materials and Methods

### Human Ovarian Biopsies

**Figure 1:**
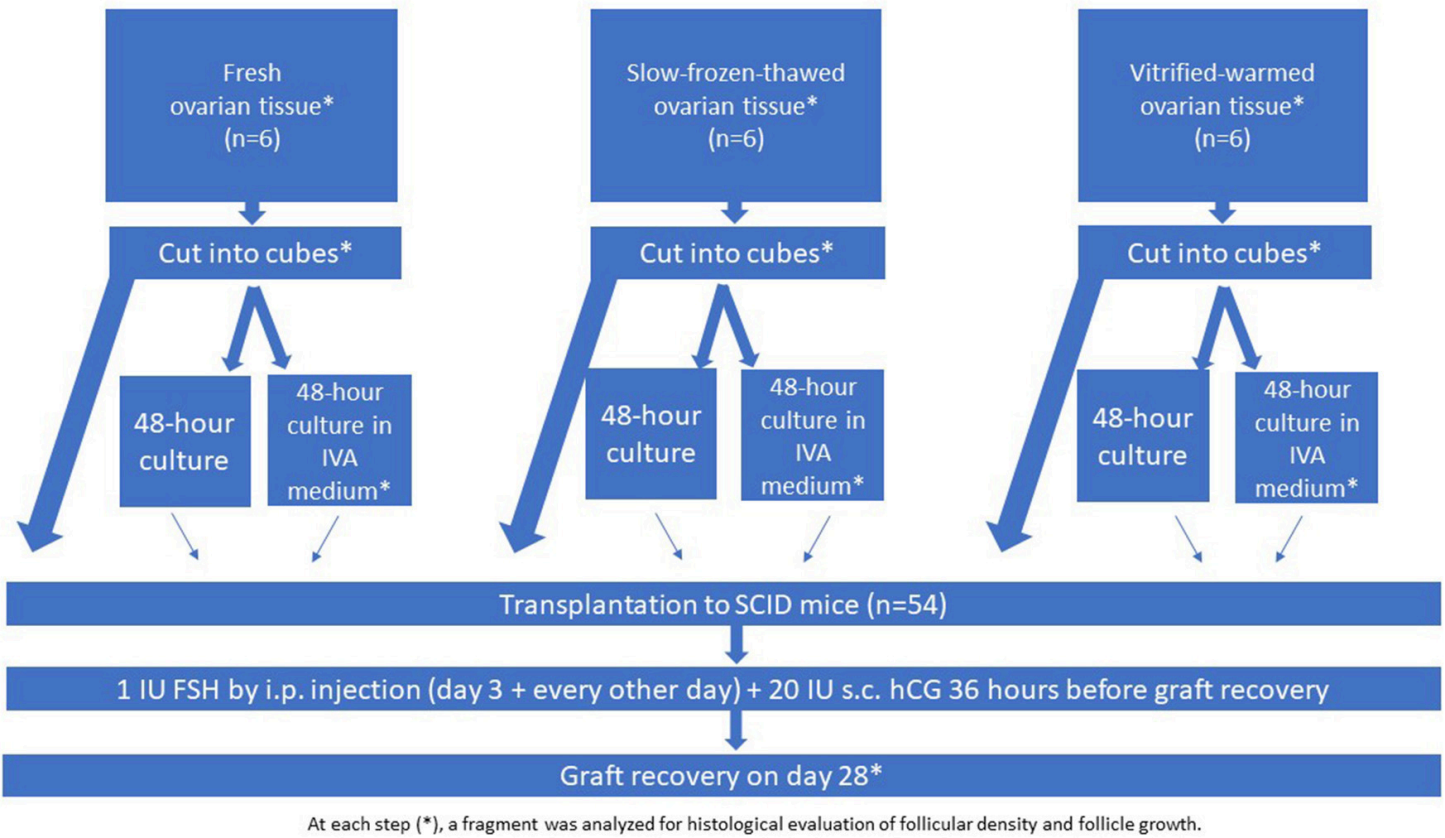
Experimental design.

Fresh and cryopreserved human ovarian tissue from young patients (*n* = 18, 26.3 ± 5.3 years of age ± range) with regular ovulatory cycles was used for this study. Use of human ovarian tissue was approved by the Institutional Review Board of the Université Catholique de Louvain (2012/23MAR/125, No. B403201213872). Ovarian tissue was taken from patients undergoing laparoscopic surgery for benign gynecological disease after obtaining written informed consent. In all cases, one piece of tissue was fixed in order to determine follicular density before any manipulation. Group I included biopsies of fresh tissue from six women (mean age: 30 y, range: 26–33 y) that were not subjected to any cryopreservation processes; group II, biopsies from six women (mean age: 20 y, range: 18–21 y) that were cryopreserved according to the slow-freezing technique; and group III, biopsies from six women (mean age: 29 y, range: 23–34 y) that were vitrified. The experimental design is presented in Figure 1.

### Cryopreservation Procedures

- After biopsy, fresh ovarian tissue pieces (measuring between 6 × 10 × 1 and 8 × 10 × 1 mm) were kept on ice in transport medium (Universal IVF Medium, Origio, Denmark), then immediately transferred from the operating room to the laboratory.
- For slow-freezing and thawing, standard protocols used in our institution to cryopreserve ovarian cortex from patients wishing to preserve their fertility were applied, as previously described (9, 10). Briefly, the ovarian fragments were transferred to cryovials containing freezing medium with dimethyl sulfoxide (DMSO, Sigma Aldrich, St. Louis, MO, USA) as a cryopotectant. The cryotubes were placed in a programmable freezer (Freeze Control CL-8800i, Cryologic, Victoria, Australia) and cooled using a slow-freezing program for ovarian tissue, before being stored at −196°C in liquid nitrogen.
- Vitrification was performed using the Ova Cryo Kit, type M (catalog VT301S, Kitazato BioPharma Co., Ltd. Fuji, Shizouka, Japan) and the Cryo Device, type M (catalog ODT × 10, Kitazato BioPharma Co., Ltd. Fuji, Shizouka, Japan). Tissue warming was achieved with the Ova Thawing Kit, type M (catalog VT302S, Kitazato BioPharma Co., Ltd. Fuji, Shizouka, Japan). Both procedures were carried out following the manufacturer's instructions.

### Xenografting

Animals welfare guidelines were followed and the protocol was approved by the Committee on Animal Research of the Université Catholique de Louvain.

Fifty-four 8-week-old female severe combined immunodeficient (SCID) mice (CB17/Icr-PrkdcSCID/IcrlcoCrl) were obtained from Charles River Laboratories (France) and left to acclimate for 1 week at the animal house facility of the Université Catholique de Louvain (Brussels, Belgium). The experiment was performed in mice aged 9–14 weeks. The animals were kept under controlled lighting conditions (13 h light/11 h dark cycles) with food and water *ad libitum*, anesthetized using ketamine (75 mg/kg; Anesketin, Eurovet, Heusden-Zolder, Belgium) and medetomidine (1 mg/kg; Domitor, Pfizer, Cambridge, MA, USA), and prepared as previously reported (11).

**Figure 2:**
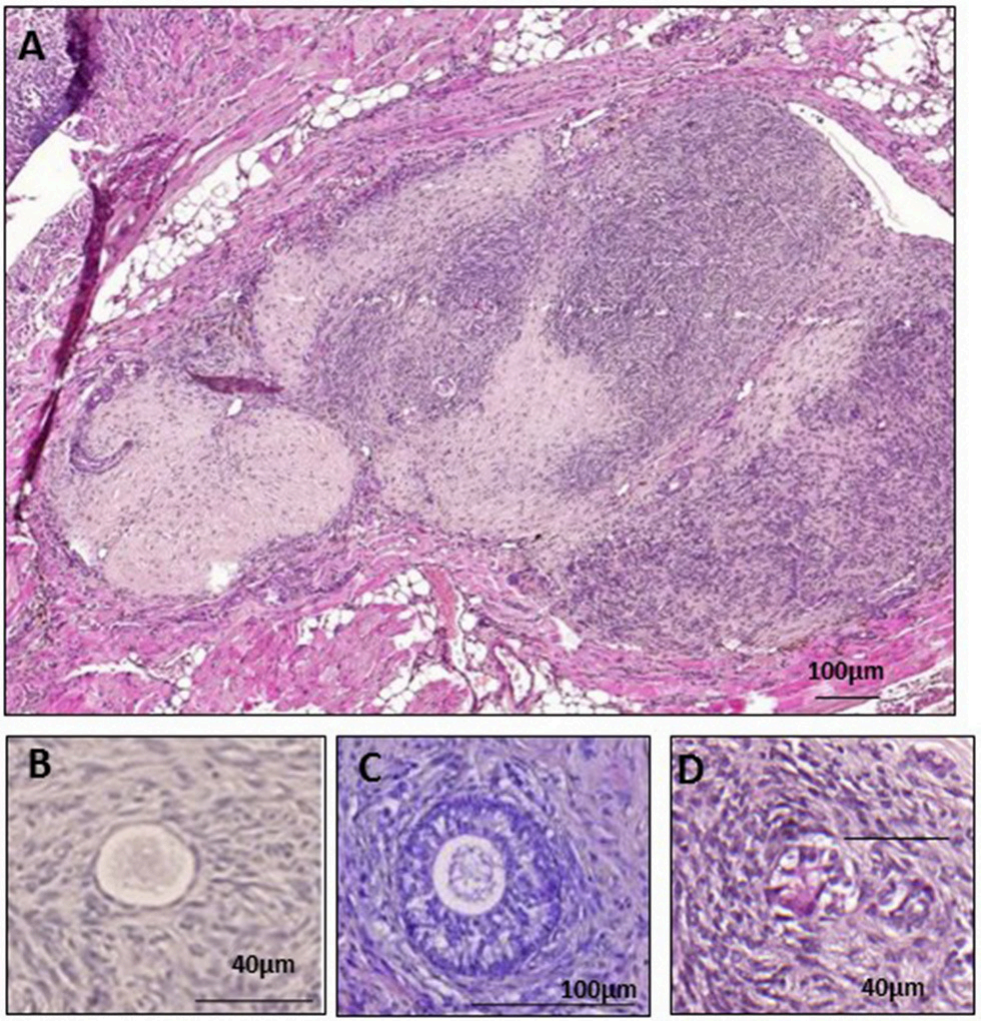
Grafted fresh ovarian tissue. **(A)** Fresh human ovarian tissue retrieved after 28 days of xenografting, surrounded by mouse tissue. Histological analysis of a primordial follicle **(B)**, a secondary follicle **(C)**, and an atretic follicle **(D)**.

The mice were placed in a ventral position, their ovaries were externalized through a 0.5 cm dorsal incision, and bilateral ovariectomy was performed. After closing the incision, they were placed in a supine position and a 2 cm ventral midline incision was made. *Two* peritoneal pockets were created ~0.5–1 cm from each side of the linea alba in the inner peritoneum using non-absorbable 6-0 Prolene suture, as described in an earlier publication (12). *Five* small cubes of human ovarian tissue were placed *one* by *one* into each peritoneal pocket, before closing tightly to avoid any loss of tissue (Figure 2A). The peritoneal layer and skin were closed using 6.0 Prolene. To reverse anesthesia, atipamezole (Antisedan, 1 mg/kg; Pfizer, Brussels, Belgium) was administered once surgery was completed. Throughout the entire surgical procedure, the animals were kept on a warming pillow until they woke up.

### Experimental Design

The grafting experiment was performed using fresh ovarian tissue (group I, *n* = 6 women), slow-frozen-thawed ovarian tissue (group II; *n* = 6 women), and vitrified-warmed ovarian tissue (group III; *n* = 6 women), assigned either for immediate grafting or culture with and without IVA prior to grafting (Figure 1). Tissue from all three groups was fragmented into small cubes (1 × 1 × 1 mm) to disrupt the Hippo signaling pathway. One-third of the ovarian cubes from each group were directly grafted to the SCID mice (without any culture), while the remaining fragments from the three groups were cultured for 48 h either in culture medium alone or in culture medium with Akt stimulators (PI3K stimulator and PTEN inhibitor) before grafting. In accordance with the previously described protocol (6), the small cubes were treated with 30 μM bpV (HOpic), a PTEN enzyme inhibitor (Merck Millipore, Calbiochem) and 150 μg/ml 740 Y-P (Tocris, Bristol, UK), a PI3K stimulator, for the last 24 h (6). Prior to transplantation, the ovarian cubes were washed three times in warmed (37°C) culture medium. All the grafts were recovered after 4 weeks (Figure 1).

Following the protocol from previous studies (2), the animals were treated on the third post-transplantation day with an intraperitoneal injection of 1 IU human FSH (hGonal, Merck Serono, Germany), which was continued every other day over the 4-week period (2, 4). Although it is probably too soon to expect to find pre-ovulatory follicles in grafted human tissue just 4 weeks after reimplantation, 20 IU human chorionic gonadotropin (hCG, Pregnyl®, Organon, the Netherlands) was injected subcutaneously 36 h before graft retrieval, in order to mimic Kawamura's protocol (2). After 28 days, the mice were euthanized by cervical dislocation and the recovered grafts were fixed in 4% (v/v) formaldehyde.

### Histological Analysis

After fixation, the biopsies were dehydrated, embedded in paraffin and serially sectioned into 5-μm-thick sections. Every fourth section was stained with hematoxylin and eosin (H&E) (Merck, Darmstadt, Germany), scanned using Mirax Scan (Zeiss, Jena, Germany) and analyzed blindly (follicle counting and classification) by two independent observers.

Surface measurements were calculated using the Mirax Viewer program. Follicles were counted and classified according to Gougeon's classification into primordial, intermediate, primary, secondary, or antral (13, 14) (Figures 2B,C). Only morphologically normal follicles were taken into account, as atretic follicles could not be classified because of their degree of degeneration (Figure 2D), and only follicles with a clearly visible nucleus were evaluated. Atresia of early follicles was diagnosed according to strict criteria (15), including eosinophilia of the ooplasm, nuclear pyknosis of GCs, cytoplasmic contraction, cytoplasmic vacuoles, and dissociation of GCs and the basal membrane (Figure 2D). The prevalence of atretic follicles was ascertained (15), and follicle density was calculated as the total number of follicles/mm^2^.

### Statistical Analysis

For intragroup analyses of follicular density, results were compared using parametric paired *t*-tests and non-parametric paired Wilcoxon tests. For intergroup analyses, non-parametric unpaired Mann-Whitney tests were applied. One-way ANOVA was carried out for follicle staging. *P* < 0.05 were considered significant.

## Results

Eighteen human samples were used for the *in vivo* experiment (Figure 1). In total, 54 mice were xenografted with human ovarian tissue placed inside specially created peritoneal pockets. On day 28, all the grafts (100%) were clearly visible and successfully recovered from all the groups.

### Follicular Density (Figure 3)

- Intragroup comparison
  - In group I (*n* = 6), ungrafted tissue had a follicular density of 1.7 follicles/mm^2^. After grafting, follicular densities were respectively, 2.79, 2.43, and 2.22 follicles/mm^2^ in the no culture, 48 h culture and 48 h IVA subgroups (*p* = 0.31).
  - In group II (*n* = 6), mean follicular density before slow-freezing was 2.15 follicles/mm^2^, and the slow-freezing procedure itself did not significantly impact this value (2.66 follicles/mm^2^ after thawing, *p* > 0.99). After grafting, follicular densities were respectively, 1.26, 0.92, and 0.66 follicles/mm^2^ in the no culture, 48 h culture and 48 h IVA subgroups, showing a significant decrease when pooled and compared to ungrafted tissue (*p* = 0.01).
  - In group III (*n* = 6), control tissues before vitrification and after warming had follicular densities of 1.13 and 1.12 follicles/mm^2^, respectively (no significant change after warming, *p* = 0.84). After graft removal, follicular densities in the three different subgroups were found to have dramatically decreased compared to ungrafted controls (*p* < 0.01), with values of 0.18, 0.04, and 0.08 follicles/mm^2^ in the no culture, 48 h culture and 48 h IVA subgroups, respectively.
- Intergroup comparison
  - In a comparison of post-grafting follicular density between groups, the slow-frozen tissue group (group II), and fresh tissue group (group I) did not show any significant difference in follicular density (*p* = 0.25, *p* = 0.39, and *p* = 0.25 for the no culture, 48 h culture and 48 h IVA subgroups, respectively).
  - On the other hand, all grafted tissues in the vitrified tissue group (group III) showed a significant decline in follicular density compared to fresh tissue (group I) (*p* = 0.05, *p* < 0.01, and *p* = 0.02 for the no culture, 48 h culture and 48 h IVA subgroups, respectively). The decrease in follicular density in group III was also significant (*p* < 0.0001) compared to group II (slow-frozen tissue), with mean follicular density in grafted vitrified tissue reaching 0.04follicles/mm^2^.

**Figure 3:**
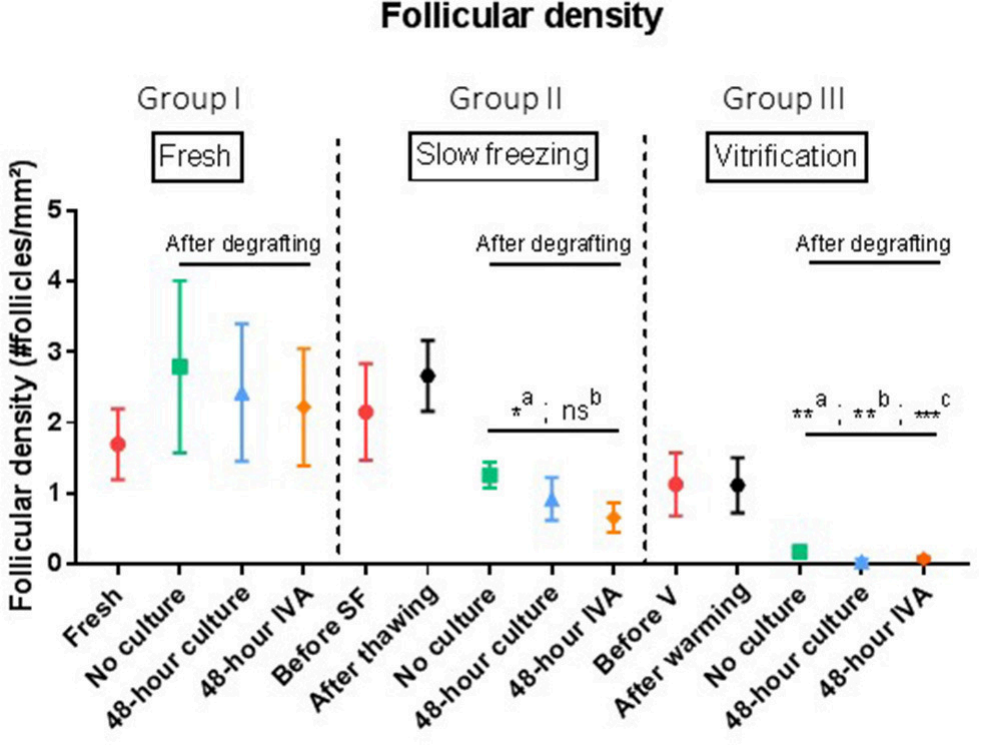
Follicular density (follicles per mm^2^). Results are expressed as mean ± SEM. Before grafting, a piece of tissue from each group (fresh, SF, and V) was kept for control purposes. For the SF and V groups, controls were also conducted after thawing or warming the tissue. Tissues from the no culture, 48 h culture and 48 h IVA subgroups were retrieved after xenografting to mice. **Intragroup comparison:** In the SF and V groups, follicle density decreased significantly after grafting (a: *p* = 0.01). However, comparison of SF tissue (group II) with fresh tissue (group 1) did not show any significant difference in follicular density (*p* = 0.25, *p* = 0.39, and *p* = 0.25 for the no culture, 48 h culture and 48 h IVA subgroups, respectively). On the contrary, grafted tissue from the V group (group III) showed a dramatic decline in follicular density compared to the fresh group (*p* = 0.05, *p* < 0.01, and *p* = 0.02 for the no culture, 48 h culture, and 48 h IVA subgroups, respectively). SF, slow-frozen; V, vitrified; IVA, *in vitro* activation. a: intragroup analysis, before vs. after grafting, b: after grafting vs. after grafting group 1, ^**^p ≤ 0.05, c: after grafting vs. after grafting group 2, ^*^p = 0.01, ^**^p < 0.01, ^***^p < 0.01.

### Percentage of Growing Follicles According to Follicle Stage (Figure 4)

In each subgroup, the percentage of primordial, intermediate, primary, secondary, and antral follicles was calculated out of total healthy follicles. In order to appreciate the effect of IVA treatment vs. no culture and 48 h culture (in culture medium alone), intermediate, primary, secondary, and antral follicles were all considered as growing follicles, and the pool of inactivated primordial follicles was compared between the three different groups.

**Figure 4:**
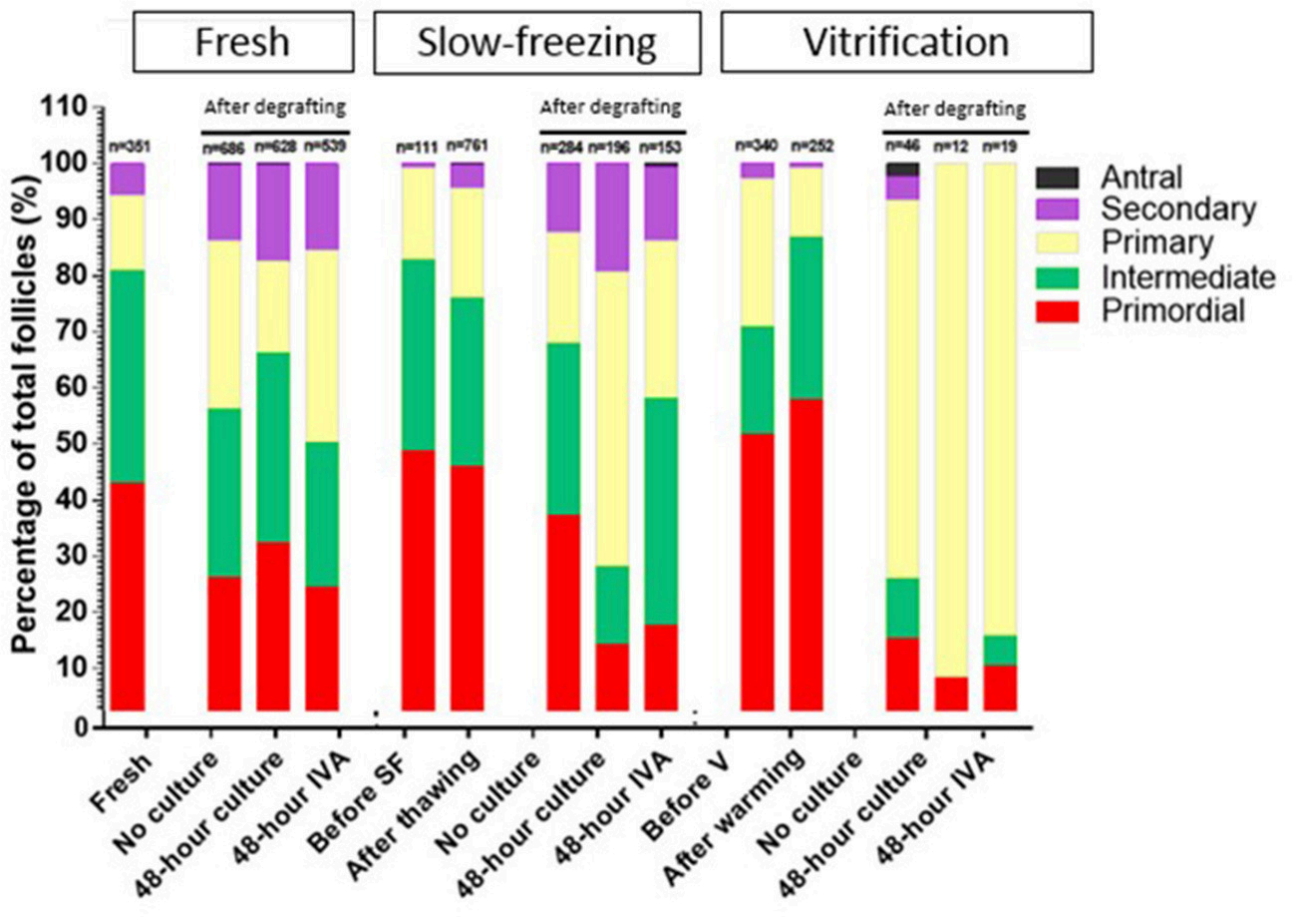
Growing follicles, defined as percentages of primary, secondary, and antral follicles out of the total number of follicles, in fresh, slow-frozen-thawed, and vitrified-warmed ovarian tissues. The percentage of primordial, intermediate, primary, secondary, and antral follicles was calculated out of total follicles for each group (fresh, slow-frozen, vitrified) and subgroup (fresh, after thawing/warming, no culture, 48 h culture, 48 h IVA). Intermediate, primary, secondary, and antral follicles were considered as growing follicles and the pool of inactivated primordial follicles was compared between the three different groups. Percentages of primordial follicles in the 48 h IVA and 48 h culture subgroups were 24.49 vs. 32.32%, *p* = 0.78, 17.65% vs. 14.29%, *p* = 0.99, and 10.53 vs. 8.33%, *p* = 0.92, for fresh, slow-frozen, and vitrified tissues, respectively.

In the fresh and slow-frozen tissue groups, 48 h of culture with culture medium alone prior to grafting did not significantly affect the proportion of primordial follicles after grafting compared to the no culture subgroup (respectively, 32.32 vs. 26.24%, *p* = 0.99; and 14.29 vs. 37.32%, *p* = 0.1). The same was true of 48 h of IVA culture (respectively, 24.49 vs. 26.24%, *p* = 0.73; and 17.65 vs. 37.32%, *p* = 0.14).

In the vitrified tissue group, 48 h of culture with culture medium alone resulted in a significant decrease in the primordial follicle pool compared to the no culture subgroup (8.33 vs. 15.22%, *p* < 0.05), but 48 h of IVA culture did not yield any significant difference (10.53 vs. 15.22%, *p* = 0.07).

Addition of PI3K stimulator and PTEN inhibitor for 48 h did not significantly change the proportion of primordial follicles in any of the groups (fresh, slow-frozen, or vitrified tissue) compared to 48 h of control culture without these molecules (respectively, 24.49 vs. 32.32%, *p* = 0.78; 17.65 vs. 14.29%, *p* = 0.99; and 10.53 vs. 8.33%, *p* = 0.92).

## Discussion

Disruption of the Hippo pathway by fragmentation of ovarian tissue, combined with stimulation of the Akt signaling pathway by treatment with PTEN inhibitors and/or PI3K stimulators, has been shown to encourage growth of preantral follicles (1, 2, 6). This combined method, known as IVA, was proposed by Kawamura and Suzuki to promote development of antral follicles in women with a diminished ovarian reserve (6, 7). These authors reported two live births issuing from vitrified tissue out of a large series of poor responders. The goal was to rapidly produce mature follicles for use in *in vitro* fertilization programs.

However, in the literature, questions have been raised about the efficacy of this IVA protocol. Indeed, our group (16), as well as that of Meirow (17, 18), both demonstrated that primordial follicle activation occurs spontaneously shortly after transplantation and is responsible for follicle loss (burn-out). This was proved by the increase in the growing follicle population and proliferation of GCs in human ovarian tissue xenografts.

Two crucial questions emerged: (1) Is promotion of follicle activation in grafts via Akt stimulators necessary, since follicle activation occurs spontaneously after transplantation? (2) As post-transplantation follicle activation (along with ischemia) could well-be responsible for follicle loss (18), should we not try (in a first step or at least very soon after grafting) to prevent this depletion by reducing activation rather than promoting it? We therefore repeated Kawamura's experimental study in an attempt to evaluate the real benefits of disrupting the Hippo signaling pathway, associated with activation of the Akt pathway, by performing 48 h culture in an IVA culture medium containing a PTEN inhibitor and PI3K stimulator, according to the protocol described by Kawamura et al. and Suzuki et al.

A) Follicular-density

Numerous studies have investigated and compared slow-freezing-thawing and vitrification-warming procedures for ovarian tissue (19, 20). Some authors consider slow-freezing to be a more promising approach than vitrification thanks to higher primordial follicle density and viability, lower levels of apoptotic cells, and a better morpho-functional status (19, 21–24). Other studies have failed to find any difference between these two cryopreservation protocols in terms of follicular density, viability of morphologically intact follicles, apoptotic cell numbers, or estradiol synthesis *in vitro* (24–29). On the other hand, a number of authors have obtained superior outcomes with vitrification with respect to GC and stromal cell ultrastructure (30–32). Fewer primordial follicle DNA strand breaks were also reported after vitrification in a review by Shi et al. (33). This lack of consistent results in the literature may be due to the heterogeneity of applied cryopreservation protocols and cryoprotectant concentrations, variety of animal models used, adequacy (or otherwise) of preparation of the grafting site, as well as different grafting durations and/or methods employed to evaluate ovarian tissue quality.

In the present study, we demonstrated that grafting vitrified ovarian tissue immediately after warming causes a more significant decline in follicular density than grafting slow-frozen tissue. Indeed, the combination of vitrification and grafting is deleterious to the post-grafting follicle reserve. Comparing follicular density in the no culture, 48 h culture, and 48 h IVA groups, we may conclude that culturing ovarian tissue for 48 h with PTEN inhibitors and PI3K stimulators before grafting is damaging to the follicle reserve after both slow-freezing and vitrification, but the decline in the ovarian reserve is significantly more pronounced after vitrification. Limitations of the present study include wide variation in the distribution and density of follicles between parts of the same ovary (34, 35), and the relatively small number of samples. Indeed, the authors were cautious in their use of human tissue, whose availability for research is limited, and biopsies of the same ovary were utilized in the different subgroups, which somewhat mitigated the chances of both beneficial and deleterious effects.

B) Follicle growth

Follicle staging allows us to calculate the percentage of growing follicles (intermediate, primary, secondary, and antral) present in each group, which is the ideal way to analyze the degree of activation. The grafting procedure yielded an increase in the percentage of growing follicles in fresh, slow-frozen-thawed, and vitrified-warmed ovarian tissue after grafting. This is consistent with our previous studies, in which both slow-frozen-thawed and vitrified-warmed human ovarian tissues were xenografted to nude mice (16, 20) and showed a high proportion of growing follicles, proving follicle activation. This follicle activation and subsequent atresia are then instrumental in the dramatic decline seen in follicular density (16), described as burn-out of the follicle pool (18, 36). The real issue was to demonstrate whether the IVA protocol could influence the maturation of follicles.

By analyzing percentages of growing follicles after culture with PTEN inhibitors and PI3K activators, and comparing them with the group cultured for 48 h without Akt stimulators, we found that numbers of growing follicles in grafts from groups I (fresh) and II (slow-frozen) were not influenced by culture with IVA. Indeed, culture for 48 h with Akt stimulators failed to elicit any significant changes, casting doubt on the need for IVA with PTEN inibitors and PI3K activators. Despite our attempts, we were unable to reproduce the results of Kawamura et al. (2) by replicating their experimental model. We applied the same IVA protocol for ovarian tissue with the same Akt stimulators, grafting human ovarian tissue for 28 days to the same mouse strain (2). The only real difference was the grafting site, namely a specially created peritoneal pocket (present study) rather than the kidney capsule (2). Although sample size was carefully computed and IVA treatment rigorously administered, no significant activation was detected in our study. One of our limitations may have been the absence of analysis of Akt, Hippo, and YAP signaling. We nevertheless feel that documenting impacts on their signaling status was not the goal of this study, as evaluating the efficacy of IVA protocols from a clinical perspective can be satisfactorily accomplished by assessing survival and growth of primordial follicles. Indeed, follicle survival is the main objective of any method of freezing and transplantation looking to preserve and restore fertility. Furthermore, Ayuandari et al. (37) recently investigated the impact of xenotransplantation on follicle recruitment and growth in frozen-thawed human ovarian tissue, and found that PTEN gene expression fell 2.47-fold after 4 weeks of xenotransplantation compared to pre-grafting controls.

Even though Kawamura et al. (2) and Suzuki et al. (6) reported two live births from vitrified tissue cultured for 48 h with Akt inhibitors, and Zhai et al. (8) one live birth from fresh tissue in a woman with a poor ovarian reserve, the total number of transplants remains unknown. These results demonstrate that the grafting procedure itself can activate the Akt pathway by reducing PTEN expression (37). Moreover, our study appears to suggest that the technique may be deleterious to the ovarian reserve, as massive follicle activation could lead to large-scale atresia. In an opinion paper, Meirow et al. question the efficacy of the IVA technique in clinical practice (38). Some *in vitro* studies have also shown that inhibition of PTEN in the human ovary results in increased activation of primordial follicles (39), but compromises development of growing follicles (40). Interestingly, in a recent review, Kawashima and Kawamura (41) described a novel infertility treatment based on mechanobiology, where they effectively abandoned the vitrification step and IVA with PTEN inhibitors and Akt stimulators. Their so-called IVA approach was limited to fragmentation of ovarian cortex into small cubes (1–2 mm), which they claimed facilitates conversion of G-actin into F-actin, leading to disruption of the Hippo signaling pathway to allow development of secondary follicles. However, we would like to stress that it is known from the literature (16, 38), as well as the present study, that activation of primordial follicles and growth to the secondary stage occur after transplantation irrespective of tissue used, be it fresh, slow-frozen, or vitrified, with or without culture with PTEN inhibitors and Akt stimulators. Moreover, Meirow et al. (38) did not observe any advantage of transplanting small pieces rather than larger pieces. In fact, no ovarian activity or follicle growth were detected in the ovary where the small fragments were grafted.

We acknowledge that follicular density was normal in our model, which is not the case in women with POI, as reported by Kawamura. While we cannot extrapolate our results to these subjects, we still have some doubts on the benefits of IVA activation with PTEN inhibitors and Akt stimulators. In our view, inhibiting the rapid growth and massive activation of primordial follicles observed after grafting of slow-frozen-thawed and vitrified tissue is one of the most important challenges we face. Such rapid growth may lead to inadequate communication between the oocyte and somatic cells, resulting in insufficient accumulation of maternal transcripts to support later embryonic development (34, 42, 43).

In conclusion, we used a xenotransplantation model to investigate whether cutting ovarian tissue into small fragments and applying IVA with PTEN inhibitors and Akt stimulators could offer added value to patients undergoing ovarian tissue cryopreservation prior to cancer treatment. We wanted to establish whether this procedure could be applied before ovarian tissue transplantation, with a view to boosting follicle growth after grafting. We were not, however, able to demonstrate any significant benefits of IVA in human ovarian tissue xenografted to SCID mice, and therefore consider it unlikely that IVA improves follicle viability.

We firmly believe that both IVA and vitrification need to be thoroughly investigated before contemplating application in a clinical setting. As our study evaluated only healthy ovarian tissue from women with a normal follicle pool, further studies should seek to repeat the same experimental protocol using tissue from individuals with a poor ovarian reserve. We feel that ongoing research should aim to prevent follicle activation, possibly by exposing ovarian tissue to media containing factors that inhibit activation prior to grafting [a role for anti-Müllerian hormone? (44, 45)], or by manipulating the Akt-FoxO and mTOR pathways (46, 47). In any case, there is still work to be done before any firm conclusions can be drawn on the value of this IVA approach.

## Data Availability

All datasets generated for this study are included in the manuscript and/or the supplementary files.

## Ethics Statement

Use of human ovarian tissue was approved by the Institutional Review Board of the Université Catholique de Louvain (2012/23MAR/125, No. B403201213872).

## Author Contributions

M-MD: conception and design of the study, experimental procedures, analysis of results, statistical analysis, and article preparation. FC: experimental procedures and analysis of results. CA: data evaluation, statistical analysis, and discussion contribution. JD: data evaluation, discussion contribution, and article revision. CV: experimental procedures, analysis of results, statistical analysis, and article preparation.

### Conflict of Interest Statement

The authors declare that the research was conducted in the absence of any commercial or financial relationships that could be construed as a potential conflict of interest.
